# A systematic review of natural health product treatment for vitiligo

**DOI:** 10.1186/1471-5945-8-2

**Published:** 2008-05-22

**Authors:** Orest Szczurko, Heather S Boon

**Affiliations:** 1 Leslie Dan Faculty of Pharmacy, University of Toronto, Canada

## Abstract

**Background:**

Vitiligo is a hypopigmentation disorder affecting 1 to 4% of the world population. Fifty percent of cases appear before the age of 20 years old, and the disfigurement results in psychiatric morbidity in 16 to 35% of those affected.

**Methods:**

Our objective was to complete a comprehensive, systematic review of the published scientific literature to identify natural health products (NHP) such as vitamins, herbs and other supplements that may have efficacy in the treatment of vitiligo. We searched eight databases including MEDLINE and EMBASE for vitiligo, leucoderma, and various NHP terms. Prospective controlled clinical human trials were identified and assessed for quality.

**Results:**

Fifteen clinical trials were identified, and organized into four categories based on the NHP used for treatment. 1) L-phenylalanine monotherapy was assessed in one trial, and as an adjuvant to phototherapy in three trials. All reported beneficial effects. 2) Three clinical trials utilized different traditional Chinese medicine products. Although each traditional Chinese medicine trial reported benefit in the active groups, the quality of the trials was poor. 3) Six trials investigated the use of plants in the treatment of vitiligo, four using plants as photosensitizing agents. The studies provide weak evidence that photosensitizing plants can be effective in conjunction with phototherapy, and moderate evidence that *Ginkgo biloba *monotherapy can be useful for vitiligo. 4) Two clinical trials investigated the use of vitamins in the therapy of vitiligo. One tested oral cobalamin with folic acid, and found no significant improvement over control. Another trial combined vitamin E with phototherapy and reported significantly better repigmentation over phototherapy only. It was not possible to pool the data from any studies for meta-analytic purposes due to the wide difference in outcome measures and poor quality ofreporting.

**Conclusion:**

Reports investigating the efficacy of NHPs for vitiligo exist, but are of poor methodological quality and contain significant reporting flaws. L-phenylalanine used with phototherapy, and oral *Ginkgo biloba *as monotherapy show promise and warrant further investigation.

## Background

Vitiligo is a hypopigmentation disorder where the loss of functioning melanocytes causes the appearance of white patches on the skin. Vitiligo affects 1% of the world population [1], but the prevalence has been reported as high as 4% in some South Asian, Mexican and American populations [2,3]. Vitiligo can develop at any age, but several studies report that 50% of cases appear before the age of 20 [4-6]. Barona found that in patients with unilateral vitiligo the mean age at onset was 16.3 years (95% CI: 12 to 19 years), compared to 24.8 years (95% CI: 22 to 28 years) in patients with bilateral vitiligo [7].

Sixteen to 35% of patients with vitiligo experience significant psychiatric morbidity [8]. Depression (10%), dysthymia (7–9%), sleep disturbances (20%), suicidal thoughts (10%), suicidal attempts (3.3%) and anxiety (3.3%) have been found in those affected with vitiligo [8]. Vitiligo can also lead to difficulties in forming relationships, avoidance of certain social situations, and difficulties in sexual relationships [9]. Vitiligo can be confused with leprosy, which also causes loss of pigment, thus further stigmatizing patients [10].

Several systematic reviews assessing the clinical efficacy of topical corticosteroids, narrowband ultraviolet light B (UVB), psoralen with ultraviolet light A exposure (PUVA), calcipotriol, l-phenylalanine, topical immunomodulators (tacrolimus and pimecrolimus), excimer laser, and surgical therapy in the management of symptoms associated with vitiligo have recently been published, including in the Journal of the American Medical Association (JAMA) and by the Cochrane Collaboration [1,11,12]. These reviews conclude that there is some evidence that topical steroids are of benefit, but report concerns with long term side effects of topical steroids. The review authors also report some evidence for the efficacy of phototherapy (UVA or UVB) as a monotherapy, or combined with psoralens or calcipotriol. However, concerns are raised about side effects such as phototoxic reactions, blistering, and lack of data on long term skin cancer risk. All three systematic reviews conclude that use of topical immunomondulators shows promise, but that further evaluation is needed. In addition, the systematic review by Whitton [1] performed for the Chochrane collaboration and the systematic review published by Forschner [11] in JAMA indicate that L-phenylalanine use with phototherapy appears promising, but that more research is necessary before firm conclusions can be drawn.

Although many clinical trials examining the use of natural health product (NHP) interventions (e.g., vitamins, minerals, herbal medicines and other supplements) for vitiligo have been published, we were unable to find any systematic reviews of these. Here we report the results of a systematic review of controlled clinical trials investigating the efficacy of NHPs in the treatment of vitiligo.

## Methods

Our objective was to complete a comprehensive, systematic review of the published scientific literature to identify NHPs that may have efficacy in the treatment of vitiligo. The databases and search terms used are listed in Table 1. All databases were searched for all available years up to August 27, 2007. The first two items (vitiligo, leucoderma) were searched individually in each database and combined together using the "OR" boolean. All search terms except the first two (vitiligo, leucoderma) were searched individually in each database and combined together using the "OR" boolean. The results from the searches of the first two bullets were then combined with the rest of the search terms by utilizing the "AND" boolean.

**Table 1:** Search Strategy

**Databases Searched **For all available years up to August 27, 2007	
All EBM Reviews (Cochrane)	International Pharmaceutical Abstracts
Allied and Complementary Medicine	Journals@ovid Full Text
CINAHL (OVID)	MEDLINE
EMBASE	Ovid Helathstar

**Search Terms**	
All items were searched individually in each database. The leucoderma and vitiligo were combined together using the OR boolean. The rest of the search group were combined together into a second group using the OR boolean. The first and second group were then combined using the AND boolean.	

• **Leucoderma**	• Inorganic chemicals
• **Vitiligo**	• Inorganic compound
• Alternative therapies	• Medicinal plant
• Amino acid	• Micronutrient
• Ayurvedic	• Mineral
• Biological factors	• Natural health product
• Biological group	• Naturopathy
• Botany	• Nutrient
• Chinese herb	• Nutrition
• Complementary medicine	• Nutrition therapy
• Complementary therapies	• Phytotherapy
• Diet therapy	• Plant
• Dietary supplement	• Plant extract
• Drugs, chinese herbal	• Plant preparations
• Essential fatty acids	• Plants, medicinal
• Ethnobotany	• Traditional medicine
• Ethnopharmacology	• Vitamin
• Herb	

The resulting list was assessed for inclusion and exclusion criteria based on title and abstract. Full reports of the studies that matched the inclusion criteria were collected, translated where necessary, and re-assessed to make a final decision regarding inclusion or exclusion. The assessment process was completed by OS, in consultation with HB.

This systematic review included prospective controlled clinical human trials. Randomization was not a requirement for inclusion in the review. Only studies which used NHPs as defined by Health Canada [13] were selected. Essentially, Health Canada defines any compound found in nature that is used for health purposes as a NHP including: vitamins, minerals, and any product derived from a plant, animal, fungi or algae. In addition, the patients in the trials must have been diagnosed with any form of vitiligo as defined by Merck [14]. The Merck manual defines vitiligo as a loss of skin melanocytes leading to sharply demarcated depigmented areas, often systematically spread. Diagnosis is made by examination, and skin lesions are accentuated under Wood's light. The inclusion and exclusion criteria were determined a priori. We included trials published in any language, treating males or females of any age and skin type. Epidemiological association studies, and publications where the full study report was not available were excluded.

The included trials were assessed for design quality using the Jadad scale [15]. Based on the Jadad scale, one point is assigned if the study is described as randomized; one point is given if the study is described as double blind, and one point is awarded if there is a description of withdrawals and dropouts in the report. Additionally, one point is awarded if the randomization method was described and is appropriate (e.g., random number tables or computer generated sequence), and one final point is awarded if the double blinding method was described and is appropriate. If the randomization or double blinding methods were inappropriate, one point for each inappropriate method is deducted. The maximum score is 5, indicating a high quality trial. Previous reviews report a mean Jadad score of 3.12 for herbal trials [16], and a mean Jadad score of 3.2 for trials of conventional medicine [17]. A study by Klassen directly comparing the quality of CAM (complementary and alternative medicine) and conventional medicine trials reports a median Jadad score of 3 for CAM and 2 for conventional medicine trials, citing no significant difference between the two [18]. These findings are consistent with another recently published study comparing the quality of placebo-controlled trials of western phytotherapy with conventional medicine, which concluded that the quality of phytotherapy trials was superior to those of conventional medicine [19].

In addition to the information required to calculate the Jadad score, the following information was extracted from each paper: study design, number of participants, compliance, inclusion and exclusion criteria, intervention, outcome measure, results and adverse events. This information was collected to facilitate comparison with the Cochrane Collaboration's review of conventional biomedical vitiligo treatments [1].

## Results

The initial search of NHP search terms and Vitiligo resulted in 986 citations. A manual review of titles and abstracts was conducted to identify studies appearing to meet the inclusion and exclusion criteria which resulted in 47 articles. The full text of these articles was obtained and reviewed. Studies without a control group, epidemiological reports, and incomplete reports were discarded. This process resulted in 15 articles that met the inclusion and exclusion criteria. Details of these studies are summarized in Table 2.

**Table 2:** Summary of Study Results

**Study ID Year & Country**	**Study Design**	**JADAD score (0–5)**	**N (n = total;** **a = active;** **c = control)**	**Dropouts**	**Inclusion/Exclusion Criteria**	**Intervention**	**Main Outcome Measure**	**Results**	
Akyol 2002 [32] Turkey	controlled	0	n = 30 a = 15 c = 15	not reported	Inclusion patients with active, extensive & generalized vitiligo 14–60 years old skin type II, III, IV	Active PUVA + vit E (900 IU/day) Control PUVA 3x/wk, 315–400 nm emission spect increased by 20% up to erythema Duration: 6 months	front and back total body photographs pigmentation rated as: - bad (0–25%) - moderate (25–74%) - good (75% or more)	**PUVA + Vit E allowed good improvement in 60% of patients, with no significant oxidation.** Active bad 3 (20%) moderate 3 (20%) good 9 (60%)	Control bad 5 (33%) moderate 4 (27%) good 6 (40%)

Bedi 1989 [26] India	placebo, controlled	0	n = 32 c = "self controlled" placebo for 1 month every 3 months	2 participants lost to follow up	Inclusion - male and female - 6–42 years old	- methoxsalen tablets 10–20 mg QD + methoxsalen ointment/lotion-0.75% + *P. kurroa *rhizomes – 200 mg dried herb BID + exposure to sunlight Duration: 36–1.8 months; 12.13 months average treatment length	response rated as: - 4 = complete cure - 3 = fast & continuous reduction in patches - 2 = slow, continues reduction in patches - 1 = v. slow progress - 0 = 0 progress	**93% responded to treatment, 27% achieved complete cure.** 4 = 8/30 3 = 17/30 2 = 2/30 1 = 3/30	

Cheng 1987 [23] China	randomized, controlled	1	n = 329 A: 16 = needle B: 9 = medication C: 5 = phototherapy D: 128 = needle + medication E: 61 = needle + medication + suntan F: 110 = needle + medication + phototherapy	not reported	not reported	- needle = 1 g/2 ml bottle, injected 2–4 mL QD - medication = 235 used "Vitiligo medication"; 48 used 8-MOP; 25 used "vitiligo cream"; rest prescribed egg yolk before suntan or phototherapy - suntan = mid-May to end of Sept whole body naked suntan, 1–3 hr per day, 8–10 am, or 4–6 pm - phototherapy = long wave phototherapy, every 2–3 days 5–25 cm2 or 1–10% of body surface	response rated as: - excellent = color of lesion turns to normal - very good = color of lesion turns normal for over 60% of body surface - good = lesion gets smaller and appearance of black granules - bad = lesion identical to that before the treatment	**Treatment with suntan effective after 60 days.** Group A: 2 = excellent 5 = very good 9 = good Group B 1 = excellent 2 = very good 6 = good Group C 4 = good 1 = bad Group F 16 = excellent 35 = very good 58 = good 1 = bad 109 = overall good result	Group D 8 = excellent 37 = very good 79 = good 4 = bad 124 = overall good result Group E 12 = excellent 26 = very good 23 = good 61 = overall good result

Cormane 1985 [20] Netherlands	controlled by run in over 4 months	0	n = 19	not reported	Inclusion - male and female - generalized vitiligo - above 18 years old Exclusion - past spontaneous improvement - CI to phenylalanine	l-phe 50 mg/kg after a low protein breakfast + UVA 2x/wk Duration: 6–8 months Controlled by run in with l-phe 50 mg/kg 2x/wk for 4 months	repigmentation was classified as dense, sparse or none	**Repigmentation achieved in 94.7% of patients.** dense repigmentation in 26.3% (5 pts) sparse 68.4% (13 pts) none 5.2 (1 pt)	

Jin 1983 [25] China	randomized, controlled	1	n = 232 A: n = 100 B: n = 51 C: n = 52 D: n = 28	not reported	not reported	A: Chinese Herb/Medicine 1 Ingredients: multi herb formula B: Corticoids 15 mg per day, oral (if effective decrease 5 mg every 2–4 weeks) C: Chinese Herb/Medicine 2 + Corticoids Ingredients different from Chinese Herb/Medicine 1* Group A,B,C also applies 30% Psoralen topically D: Control apply 30% Psoralen topically Duration: 2 months	response rated as: - Excellent = all lesions disappear, normal skin color reappears - Very good = reduction in lesion, >60% of damaged skin surface regains normal skin color - Good = reduction in lesion, 10–60% of damaged skin surface regains normal skin color - Bad = lesion shows no change in appearance or size, <10% of damaged skin surface regains normal skin color	**Chinese medicine 2 & corticoids provided the best treatment.** Group A 13% = excellent 17% = very good 34% = good 36% = bad Group C 31% = excellent 14% = very good 28% = good 27% = bad	Group B 18% = excellent 13% = very good 29% = good 40% = bad Group D 0% = excellent 6% = very good 27% = good 67% = bad

Khemis 2004 [30] France	randomized, double blind	2	n = 30 self controlled by contralateral side	13 dropped out	Inclusion participants with 2 vitiliginous lesions each with at least 10 cm in diameter Exclusion unstable vitiligo	Active Vitix + UVB Control Placebo + UVB	primary: - reduction of lesion surface area by 50% - photographs under normal and Woods Lamp	**The difference between control and active was not significant** 4 participants did not repigment 4 participants received a reduction 6 achieved a reduction of more than 50% of lesion with Vitix and UVB but not with control 3 achieved had a reduction of more than 50% of lesion with control but not with Vitix and UVB.	

Liu 2003 [24] China	randomized, controlled	1	n = 74 a = 41 c = 33	not reported	not reported	Active - Xiaobai mixture orally (30 g walnut, 10 g red flower, 30 g black sesame, 30 g black beans, 10 g zhi bei fu ping, 10 g lu lu tong, 5 plums)1 mL is equivalent to 0.1 g raw medication, 160 mL once a day Control - 10 mg 8-MOP TID Duration: 3 months	repigmentation rated as: - Excellent = normal skin color reappears - Very good = >50% of damaged skin surface regains normal skin color - Good = 10–50% of damaged skin surface regains normal skin color - Bad = <10% of damaged skin surface regains normal skin color	**95% of active and 79% of control group showed good results.** Active - 12 = excellent results - 16 = very good results - 11 = good results - 2 = bad results - Overall 95.12% showed good results Control - 3 = excellent results - 14 = very good results - 9 = good results - 7 = bad results	

Middelkamp-Hup 2007 [28] Netherlands	placebo controlled, randomized, double blind	5	n = 50 a = 25 c = 24	1 lost to follow up at last session	Inclusion - 18 years and older - vitiligo vulgaris Exclusion - history of skin cancer - photosensitive - pregnancy or lactation - segmental vitiligo - phototherapy 3 months prior - use of topical treatments - starting vitamins during the study	Active *Polypodium leucotomos *250 mg TID + NB-UVB 2x/wk (210–360 j/cm2 at start & gradually increased) Control placebo TID + NB-UVB Duration: 25–26 weeks	severity of vitiligo rated as: - very severe - severe - more severe - less severe - not so severe secondary measures: - digital photography of all vitiligo lesions - patient self assessment scale 0–10 - monitored at w0, w6, w12, w26 - for quality of life used skin index-29	**Clear trend toward increase in repigmentation in head and neck area with NB-UVB and oral *P. leucotomos *treatment.** Percent Repigmentation : Head & Neck active = 44% control = 27% Extremities active = 30% control = 26%	Trunk active = 36% control = 30% Hands and Feet active = 24% control = 19%

Parsad 2003 [2] India	randomized, placebo controlled, double blind	2	n = 52 a = 26 c = 26	1 active 2 control "withdrew for reasons unrelated to the study"	Inclusion gradually progressive slow spreading vitiligo Exclusion more than 3 lesions or surface area of depigmentation greater than 10 cm2 over last 1 month	Active 40 mg *Ginkgo biloba *with 9.6 mg ginkgoflavone glycosides, TID Control sugar capsule TID Duration: 6 months	- photographs at 6-weekly intervals - repigmentation judged as minimal (25%), moderate (50%), marked (75%), complete (100%)- degree of repigmentation was determined by comparison of paper tracings, written descriptions, and actual measurement of vitiliginous areas	***Gingko biloba *was significantly more effective.** Active -20 pts stopped progression of disease (out of 25) - 10 pts showed marked to complete repigmentation (75–100%) (out of 25) Control - 8 pts arrested progression of disease (out of 22) - 2 pts showed marked to complete repigmentation (75–100%) (out of 22)	

Rojas-Urdaneta 2007 [22] Venezuela	randomized, double blind	3	n = 100 divided into 5 groups	all completed the study	Inclusion - stable vulgar vitiligo - no treatment for 5 months before study - 18 to 50 years old - male or female - no other pathology - provided consent for study and treatment Exclusion - concomitant disease - treatment of vitiligo 5 months prior to study - not complying with instructions	A: antioxidant and mitochondrial stimulating cream & oral antioxidants & phenylalanine B: placebo cream and oral antioxidants and phenylalanine C: oral administration of antioxidants and phenylalanine D: placebo cream E: antioxidant and mitochondrial stimulating cream cream = VitilVenz phenylalanine = 500 mg q12 hrs for 5 months oral antioxidants = Vit A 20,000 IU Vit C 1000 mg Vit E 400 IU Zinc 15 mg Selenium 50 μg Magnesium 2 mg CoQ10 75 mcg pygnogenol 1 mg	main outcome measures: - clinical area of newly formed pigment every 30 days - point system for classifying size - histological presence of melanocytes at beginning and end	**The use of antioxidant and mitochondrial cream, and oral antioxidants and phenylalanine provided best results.** group A - best results, 10.4 points (p < 0.001) group E - second best results 9.77 points (p < 0.001) group B, C - 4 points (p < 0.05) group D - comparison placebo group	

Siddiqui 1994 trial 1 [21] Netherlands	randomized, controlled, open label	0	n = 149 A: no tx = 11 B: l-phe +UVA = 132 C: l-phe alone = 6	out of 132 l-phe + UVA, 60 dropped out because they were unable to attend every 3 months for evaluations)	Inclusion - disseminated vitiligo over 10–40% of body - age 18–61 yrs Exclusion - only distal vitiligo - only acrofacial vitiligo - contraindications (same as Carmane 1985) - patients in the open trial were not enrolled in the blind trial	A: l-phe 50–100 mg/kg + UVA 2x/wk; 30–45 min after l-phe ingestion daily B: l-phe 100 mg alone C: no tx Duration: 1.5 yrs	length & width measures + color photographs every 3 months Repigmentation Classification: - partial 25–40% - incomplete 40–60% - good 60–80%	**L-phenylalanine + UVB provided better results than L-phenylalanine alone.** Group A positive 94 stable 20 deterioration 18 n = 132 Group C positive 0 stable 7 deterioration 4 n = 11	Group B positive 0 stable 5 deterioration 1 n = 6

Siddiqui 1994 trial 2 [21] Netherlands	placebo controlled, randomized, double blind	3	n = 32	5 in placebo 3 active (2 stopped for personal reasons)	Inclusion 18–56 years old vitiligo for 1–33 years	l-phe 100 mg/kg/day or placebo UVA 2–3x/wk or no irradiation Duration: 6 months	length and width measured + color photographs every 3 months Repigmentation Classification: - partial 25–40% - incomplete 40–60% - good 60–80%	**L-phenylalanine + UVB provided best results.** l-phe + UVA positive 6 stable 1 deterioration 1 % repigmentation 30–60 n = 8 dropouts 0 l-phe positive 1 stable 3 deterioration 1 % repig 25 n = 5 dropouts 3	placebo + UVA positive 0 stable 4 deterioration 2 % repig 0 n = 6 dropouts 2 placebo positive 0 stable 3 deterioration 2 % repig 0 n = 5 dropouts 3

Tjioe 2002 [31] Sweden	randomized, open label, controlled	2	n = 27 a = 14 c = 13	1 active and 1 control did not repigment more than 5% after 4 months and were advised to stop	Inclusion - male & female over 18 yrs - stable vitiligo vulgaris (1 year with no changes) - fitzpatrick's skin II – IV Exclusion - other vitiligo treatment - history of skin cancer - on photosensitizing medications - Psychiatric/epileptic disorders - renal failure or allergies to substances in trial	Active - UVB 3x/wk - oral cobalamin 1000 ug sustained release BID - folic acid 5 mg BID Control - UVB (311 nm) 3x/wk, started at 0.10 J/cm2 increased by 0–30% on individual basis Duration: 12 months	primary: visually scored as percentage of repigmentation of depigmented lesions primary criterion were areas showing most active repigmentation secondary: before and after photographs with % repigmentation visually estimated	**No significant difference between the two groups.** 25 out of 27 total showed prominent repigmentation in some areas, 1 in each group did not respond more than 5%	

Valkova 2004 [27] Bulgaria	controlled (group assignment by alternation)	0	n = 33 a = 16 c = 17	not reported	Inclusion -vitiligo - male & female and 8 kids - age 6–59 (mean 24.3 years) - photo-type II-IV - 2–21 years of disease	Active - local KUVA - 5% khellin in water/oil applied to lesion area - 1 hr later – UVA – 2–2.5 j/cm2 Control - systemic PUVA - oral psoralen (0.4 mg/kg) - 2 hrs later-UVA – 1–1.5 j/cm2 - 3x/wk Duration: 4.1 months	- % of repigmentation measured by planimetry (actual measurement)- % repigmentation according to rule of three	**Both KUVA and PUVA treatment lead to similar results, but KUVA is local, therefore with potentially with less adverse events.** active n 90–100% 3(18.8%) 60–80% 4(25.0%) 20–50% 7(56.2%) no effect 2 Signs of repigmentation appeared between 10–16 procedures	control n 90–100% 2 (11.8%) 60–80% 7 (41.2%) 20–50% 7 (41.2%) no effect 1

anonymous 2006 [29] Japan	randomized, double blind	0	n = 19 a = 10 c = 9	not reported	Inclusion generalized vitiligo Exclusion acral or segmental vitiligo	Active PUVA + *Polypodium leucotomos* Control PUVA + placebo Duration: 12 wks	repigmentation scored as: - none or minimal (<25% repigmentation) - mild (25–50%) - moderate to excellent (>50%)	**Percentage of patients with skin repig. greater than 50% was significantly higher in PUVA + *Polypodium*** active none = 2 mild = 3 moderate = 5	Control none = 5 mild = 4 moderate = 0

Of the 15 trials included in this review 11 were published in English, three in Chinese, and one in French. Trial reports published in Polish, Russian, German, Spanish and Italian were reviewed during the second stage of data collection; however, these were either incomplete, did not involve NHPs, or were uncontrolled and therefore did not meet our inclusion criteria. Of the trials included in this review, ten studied NHPs as an adjunct to UVA or UVB, and five investigated NHPs as the primary treatment. We group the clinical trials into four broad categories of NHP interventions: L-phenylalanine, traditional Chinese medicine (TCM) products, herbs, and vitamins.

The most commonly studied intervention was L-phenylalanine, assessed in three trials as an adjuvant to UVA or UVB phototherapy [20,21] (Siddiqui references two trials in one paper), and in one trial with other agents but without phototherapy [22]. At doses ranging from 50 mg/kg to 100 mg/kg for 6 to 18 months, all trials reported beneficial effects. The studies recruited from 19 to 149 patients. Two of the studies had a Jadad score of 0, and two had a Jadad score of 3. The main problems were lack of randomization and poor control in two studies, high participant dropout in another trial, and inconsistent outcome measures. It is not possible to pool the data from the studies for meta-analytic purposes due to the wide differences in outcome measures. Overall there is moderate evidence that L-phenylalanine has efficacy as an adjunctive agent to phototherapy. Additional controlled research that focuses on L-phenylalanine in conjunction with UVA or UVB phototherapy is necessary to confirm these preliminary findings.

Three trials used traditional Chinese medicinal herbs for the treatment of vitiligo [23-25]. Each clinical trial used different remedies, some given by themselves, or in conjunction with phototherapy or sun tan advice. Specifically, Liu used a Xiaobai mixture containing walnut, red flower, black sesame, black beans, zhi bei fu ping, lu lu tong, and plums; details of the products investigated were not provided for the other two trials. All three trials compared the NHP intervention to conventional biomedical treatments of vitiligo (phototherapy, corticosteroids, or psoralen) in the control group. The studies ranged from 74 to 329 patients and 2–3 months. Even though two of the studies are relatively large (329 and 232 participants), participants were divided into multiple groups. No further details of group allocation are provided. All three trials indicate positive results for the NHP intervention. All three trials received a Jadad score of 1. Varying and poorly described treatments, small treatment group size, and inconsistent outcome measures were the main problems with the studies. It is not possible to pool the data from the studies for meta-analytic purposes due to the varying treatments and differences in outcome measures. Overall, there is weak evidence that some traditional Chinese medicinal herbs may be useful for the treatment of vitiligo. None of the treatments' effects have been replicated and overall the studies are of poor methodological quality.

Six trials investigated the use of plants in the treatment of vitiligo. Four of these trials utilized plants as photosensitizing agents (*Picorrhiza kurroa*, a khellin extract, and two *Polypodium leucotomos*) [26-29] all given orally in conjunction with UVA or UVB phototherapy; one investigated the use of oral *Ginkgo biloba *by itself (40 mg TID) [2]; and one investigated the topical use of an extract of *Cucumis melo *[30]. Treatments lasted from 3 to 12 months, and the studies were small, ranging from 9 to 50 participants. Only one trial by Middelkamp-Hup utilising *Polypodium leucotomos *was of good quality, scoring 5 on the Jadad scale, while the quality of the other four trials was poor, reflected by Jadad scores ranging from 0 to 2. The main problems with the studies were the small sample size, poor study design, and inconsistent outcome measures. Overall, there is weak evidence that photosensitizing plants can be effective in conjunction with phototherapy, and moderate evidence that *Ginkgo biloba *by itself can be useful for vitiligo. *Cucumis melo *treatment was not found to be statistically significant over placebo cream. Additional research to confirm these preliminary trials is necessary.

Two trials investigated the use of vitamins as adjuvants to UVA or UVB phototherapy [31,32]. Oral cobalamin (1000 mcg BID) and folic acid (5 mg BID) were given in one trial, and oral vitamin E was given in another (900 IU, type of vitamin E not specified). After 12 months of treatment the cobalamin and folic acid study reported no significant difference compared to a phototherapy control. On the other hand, 6 months of treatment with combined vitamin E and phototherapy achieved significantly better repigmentation than phototherapy only. The studies were small, ranging from 27 to 30 participants, and neither had a Jadad score greater than 2. There was a lack of statistical information, and inconsistent outcome measures were used. Overall, there is no evidence for using oral cobalamin and folic acid with phototherapy for the treatment of vitiligo, while the evidence for vitamin E as an adjunct to phototherapy is weak.

Only five trials discussed adverse events, the most common of which were erythema, pruritis, and nausea. Erythema was reported in studies utilizing phototherapy. Pruritis was reported in trials using metoxsalen with *Picorrhiza kurroa *and *Polypodium leucotomos *with phototherapy. Nausea and gastrointestinal complaints were reported in trials utilizing *Ginkgo biloba*, *P. leucotomos *with phototherapy, and Vitamin E with phototherapy. It is difficult to ascertain whether the NHPs or their concomitant treatments caused these adverse reactions. All reported adverse reactions were minor. Most studies did not adequately report adverse events, and the small sample size of the trials makes generalizations difficult. A complete list of adverse events reported is in Table 3.

**Table 3:** Adverse Events

Study ID	Intervention	Adverse Events
Akyol 2002 [32]	vit E + UVA vs UVA	erythema, nausea, headaches in both groups
Bedi 1989 [26]	methoxsalen + *Picorrhiza kurroa* vs methoxsalen	pruritis in "some cases", "no long term side effects"
Middelkamp-Hup 2007 [28]	*Polypodium leucotomos *+ UVB vs UVB	mild transient itching (10 active, 5 control); dryness of skin (5 active, 3 control); mild gastrointestinal complaints (4 active, 5 control)
Parsad 2003 [2]	*Ginko biloba* vs placebo	2 subjects experienced nausea
Tjioe 2002 [31]	UVB+cobalamin+folic acid vs UVB	transient erythema in all patients within 24 hrs of phototherapy, prickling sensation at site of erythema

## Discussion

Most of the clinical trials identified were uncontrolled, and thus could not be included in this review. Of the controlled clinical trials reviewed, most were of poor methodological quality with only one trial receiving a Jadad score of four or greater, and 13 receiving a score of three or less. Only five trials could be classified as randomized controlled trials. We found that most of the studies were poorly reported, often lacking information on dosage frequency, dosage strength, participant withdrawal, statistical analyses and randomization. Few were double blind, and there is no mention of compliance in any report. These findings are consistent with the poor quality of vitiligo trials of conventional biomedical treatments, as discussed in the systematic review by Whitton [1] for the Cochrane collaboration.

Whitton [1] also expressed concern with the lack of common, reliable outcome parameters and the variation of methods for scoring repigmentation. Our findings mirror this problem. We found that most NHP trials set several repigmentation ranges and measured the number of participants falling into each category at the end of treatment. These repigmentation ranges seem to be arbitrary and vary between trials, making data pooling and comparing results across treatments difficult.

Further clinical research in the treatment of vitiligo is necessary. Two areas are particularly intriguing. First, well designed clinical trials should attempt to replicate the studies utilizing L-phenylalanine in conjunction with phototherapy treatment. Several small clinical trials published so far provide positive results consistently with replication, but larger more definitive trials are necessary. Second, the use of *Ginkgo biloba *alone for the treatment of vitiligo holds potential promise. The use of *Gingko biloba *without phototherapy is likely to avoid the adverse reactions and unknown long term risks associated with phototherapy. If effective, *Ginko biloba *would also be a less costly and easier treatment for vitiligo. Parsaud's trial is methodologically sound, and provides promising results. However, one published trial is not enough evidence to impact clinical practice. The need to find a safe and effective treatment is particularly important with vitiligo, where up to 50% of cases develop in the paediatric population; at a time when the condition has the greatest impact on psychological development.

The poor quality of vitiligo trials has been recognized. Several validated vitiligo outcome measures have recently been suggested and should be utilized in future clinical trials. One such measure is the Vitiligo Area Scoring Index (VASI) which takes into account both the lesion area and the intensity of depigmentation [33]. An even more precise measurement of vitiligo has been described by the Vitiligo European Task Force [34]. The first component summarizes relevant disease and patient information, while the second component is an assessment system evaluating the extent of depigmentation using the rule of 9's. (The rule of 9's is a quick way of estimating the affected skin's surface area, where the palm is 1% of the surface area; arms, and face & scalp are each 9% of the surface area; and legs, the back, and front are 18% each). In addition, the disease is staged based on cutaneous and hair pigmentation of the largest lesion in each body area, and ranked on a scale from 0–4. Assessment of spreading is based on a Wood's lamp examination of the largest lesion in each body area. The clinical use of this system has been evaluated by several European University clinics, and has showed good internal reliability [34]. Future trials should also differentiate and evaluate vitiligo treatments among their ability to induce stability, thus arresting further depigmentation, and repigmentation.

Our review is limited by the poor quality of the clinical trials. While there are many published clinical trials using NHPs for the treatment of vitiligo, there is a lack of randomized clinical trials. The different NHPs tested, and the variability of the outcome measures make it impossible to pool any data. Many trials seem to be explorative trials, with no published follow up investigations replicating the findings.

## Conclusion

Reports investigating the efficacy of NHPs for vitiligo exist, but are of poor methodological quality and contain significant reporting flaws. Most trials use NHPs as an adjunct to UVA or UVB phototherapy. There are few controlled trials assessing efficacy of NHPs for vitiligo, but those that have been published generally show weakly positive outcomes with few adverse reactions. L-phenylalanine used with phototherapy shows promise. The use of oral *Ginkgo biloba *as monotherapy for vitiligo is also promising. Ginkgo's apparent efficacy without the need for phototherapy, thus eliminating the adverse events inherent with phototherapy make it a therapeutic option worth investigating. Further high quality investigations into the use of NHPs in the treatment of vitiligo, particularly L-phenylalanine and *Ginkgo biloba *are needed.

## List of Abbreviations

CAM: complementary and alternative medicine; JAMA: the Journal of the American Medical Association; NHP: natural health product; PUVA: psoralen with ultra violet light A; TCM: traditional Chinese medicine; UVB: ultra violet light B.

## Competing interests

The authors declare that they have no competing interests.

## Authors' contributions

OS conceived of the study, carried out the data collection and review, drafted the manuscript and approved the final submitted version. HSB conceived of the study, guided the design, helped to resolve methodological concerns, critically revised the manuscript and approved the final submitted version.

## Pre-publication history

The pre-publication history for this paper can be accessed here:


